# Supplementary material for: Multiphoton excited singlet/triplet mixed self-trapped exciton emission
Source: Nat Commun. 2023 Mar 10;14:1310. doi: 10.1038/s41467-023-36958-3 (PMC10006417; doi:10.1038/s41467-023-36958-3)
Supplement: Supplementary file 1 — Supporting information file [file 41467_2023_36958_MOESM1_ESM.docx]

**Supplementary information**

**Multiphoton Excited Singlet/Triplet Mixed Self-Trapped Exciton Emission**

**Rui Zhou^1,7^, Laizhi Sui^2,7^, Xinbao Liu^3^, Kaikai Liu^1,✉^, Dengyang Guo^1,4^, Wenbo Zhao^1^, Shiyu Song^1^, Chaofan Lv^1^, Shu Chen^1^, Tianci Jiang^5,6^, Zhe Cheng^5,6^, Sheng Meng^3^, Chongxin Shan^1,✉^**

*^1^Henan Key Laboratory of Diamond Optoelectronic Materials and Devices, School of Physics and Microelectronics, Zhengzhou University, Zhengzhou, P. R. China.*

*^2^State Key Laboratory of Molecular Reaction Dynamics, Dalian Institute of Chemical Physics, Chinese Academy of Sciences, Dalian, P. R. China.*

*^3^Institute of Physics, Chinese Academy of Sciences, Beijing, China*

*^4^Department of Physics, Cavendish Laboratory, University of Cambridge; Cambridge, UK.*

*^5^Department of Pulmonary and Critical Care Medicine, The First Affiliated Hospital of Zhengzhou University, Zhengzhou, P. R. China.*

*^6^Henan Key Laboratory for Pharmacology of Liver Diseases, Zhengzhou, P. R. China.*

*^7^These authors contributed equally to this work.*

*^✉^e-mail:* [*liukaikai@zzu.edu.cn*](mailto:liukaikai@zzu.edu.cn)*;* [*cxshan@zzu.edu.cn*](mailto:cxshan@zzu.edu.cn)*.*

**Contents**

[Figure S1 3](#_Toc126593542)

[Figure S2 3](#_Toc126593543)

[Figure S3 4](#_Toc126593544)

[Table S1 4](#_Toc126593545)

[Figure S4 5](#_Toc126593546)

[Figure S5 5](#_Toc126593547)

[Table S2. 6](#_Toc126593548)

[Figure S6 7](#_Toc126593549)

[Figure S7 7](#_Toc126593550)

[Figure S8 8](#_Toc126593551)

[Table S3 8](#_Toc126593552)

[Table S4 8](#_Toc126593553)

[Figure S9 9](#_Toc126593554)

[Figure S10 9](#_Toc126593555)

[Figure S11 10](#_Toc126593556)

[Figure S12 11](#_Toc126593557)

[Figure S13 12](#_Toc126593558)

[Figure S14 13](#_Toc126593559)

[Figure S15 14](#_Toc126593560)

[Figure S16 15](#_Toc126593561)

[Figure S17 15](#_Toc126593562)

[Figure S18 15](#_Toc126593563)

[Figure S19 16](#_Toc126593564)

[Table S5 16](#_Toc126593565)

[Table S6 17](#_Toc126593566)

[Table S7 17](#_Toc126593567)

[Figure S20 18](#_Toc126593568)

[Figure S21 20](#_Toc126593569)

[Figure S22 21](#_Toc126593570)

[Figure S23 22](#_Toc126593571)

[Figure S24 23](#_Toc126593572)

[Table S8 23](#_Toc126593573)


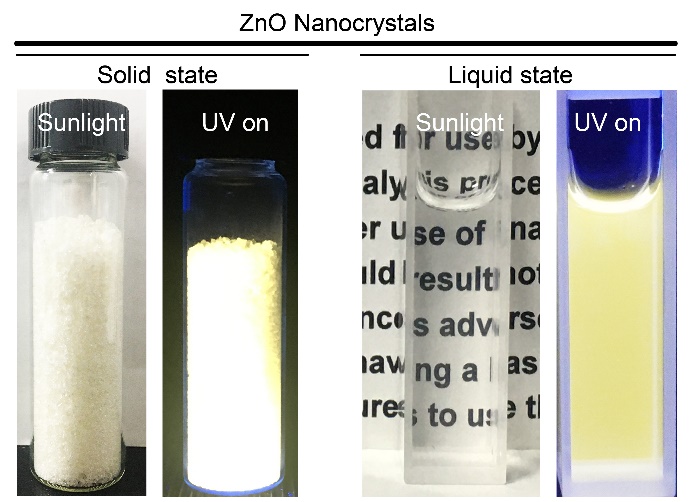


Figure S1**|** Digital photos of ZnO nanocrystals in the solid (left) or liquid state (right) under the sunlight and UV light excitation.


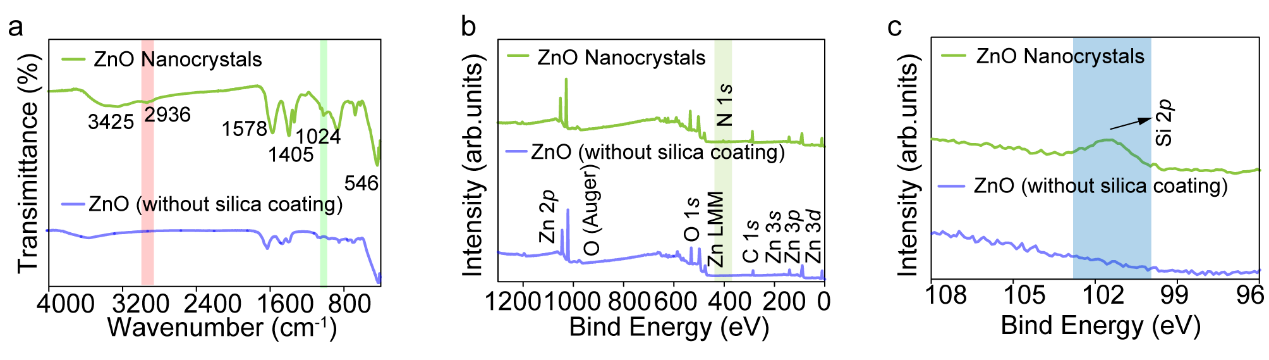


Figure S2**|** **a**, FTIR spectra of ZnO nanocrystals with and without silica coating. **b**, XPS spectra of ZnO nanocrystals with and without silica coating. **c**, Si 2*p* XPS spectra in ZnO nanocrystals with and without silica coating.

As shown in Fig. S2a, the new peak at 2936 and 1024 cm^-1^ appeared in the FTIR spectrum of ZnO nanocrystals and were attributed to the characteristic stretching vibration of C-H and Si-O. And the peak intensity of the N-H stretching (3425 cm^-1^) and bending (1578 cm^-1^) vibration, and the C-H bending (1405 cm^-1^) vibration in ZnO nanocrystals were significantly higher than those of the corresponding vibration in ZnO (without silica coating), which might also due to the silicon coating. In addition, XPS spectra clarified that N and Si element did not arise in the ZnO nanocrystals (without silica coating) (Fig. S2b and S2c). The above results confirmed that ZnO nanocrystals with good water soluble were successfully prepared.


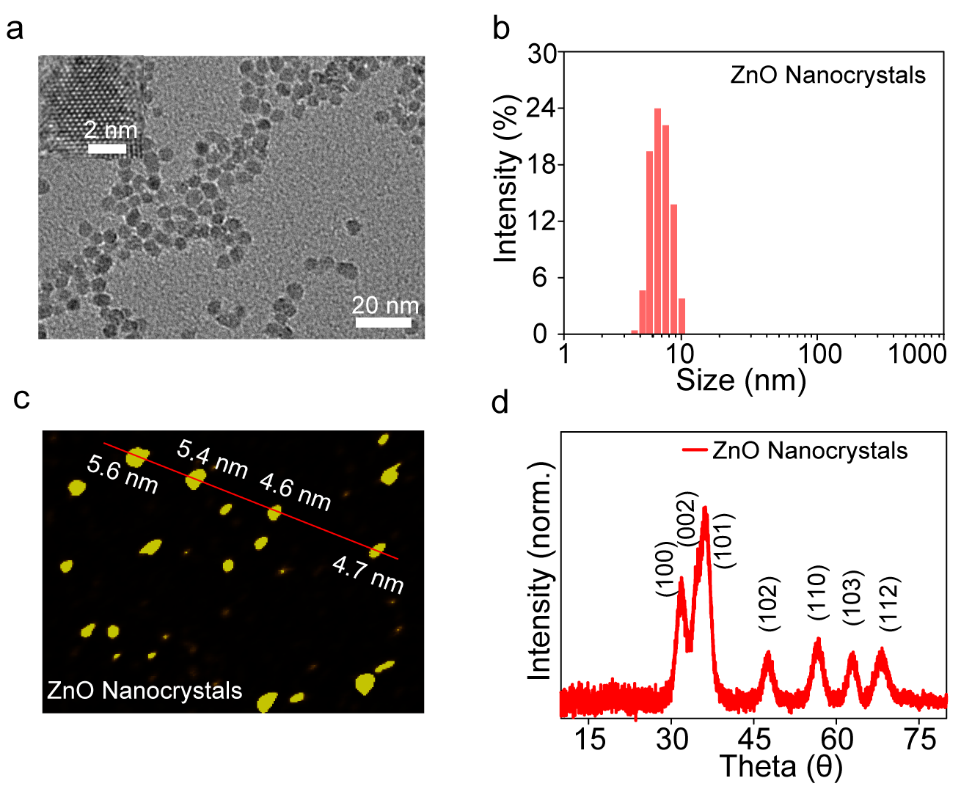


Figure S3**| a**, TEM image of ZnO nanocrystals (inset: high-resolution TEM image). **b**, Dynamic light scattering spectrum of ZnO nanocrystals. **c**, AFM image of ZnO nanocrystals. **d**, XRD pattern of ZnO nanocrystals.

Table S1. The ratio of Zn to O atoms in three ZnO samples without silica coating (ZnO nanocrystals 1, ZnO nanocrystals 2 and ZnO nanocrystals 3) by the XPS measurement.

| Sample | The Zn/O values | The average Zn/O values |
| --- | --- | --- |
| ZnO nanocrystals 1 | 0.92 | 0.99 |
| ZnO nanocrystals 2 | 1.00 |  |
| ZnO nanocrystals 3 | 1.04 |  |


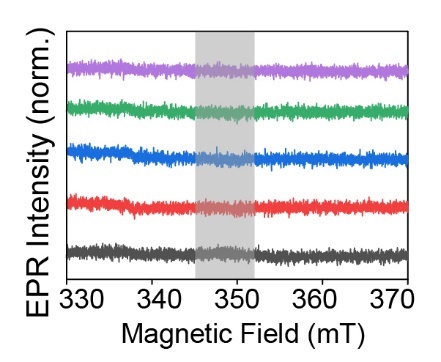


Figure S4**|** The EPR spectra of ZnO nanocrystals tested for five times at room temperature.


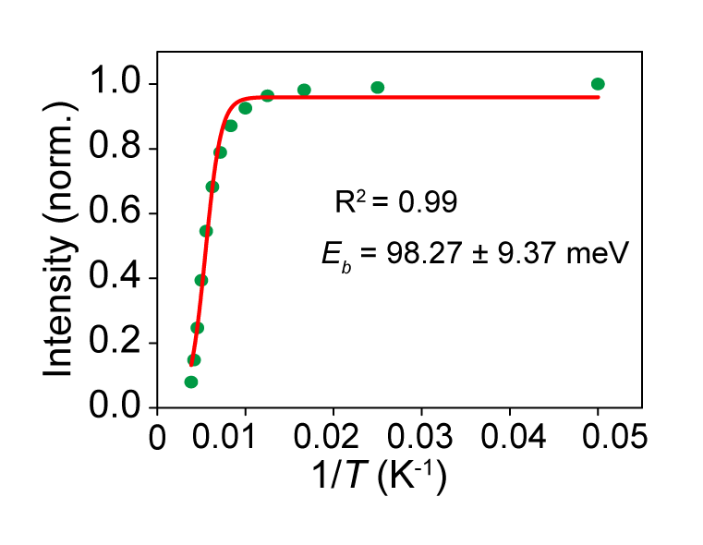


Figure S5**|** Temperature-dependent integrated STE emission intensity of ZnO nanocrystals (20 K-260 K).

Clearly, the integrated STE emission intensity monotonically increased as the samples were cooled down because the defects that lead to nonradiative recombination loss at elevated temperature are deactivated at low temperatures, based on the following equation:

*I_T_=*${I_{0}}/{(1+Aexp\left( -\frac{E_{b}}{k_{B}T} \right))}$ (S1)

where *I_0_* and *I_T_* are the emission intensity at 0 K and T K, respectively; *A* is a constant, *E_b_* is the binding energy, *k_B_* is Boltzmann constant.

Table S2. Huang−Rhys factor *S* of different materials.

| Material | Huang−Rhys factor *S* | Reference |
| --- | --- | --- |
| CdSe | 1 | 1 |
| ZnSe | 0.3 | 2 |
| CsPbBr_3_ | 3.2 | 3 |
| Cs_2_NaYCl_6_ | 7 | 4 |
| AgCl:Br | 22 | 5 |
| Cs_2_AgInCl_6_ | 38.7 | 6 |
| Cs_2_Ag_0.60_Na_0.40_InCl_6_ | 40.9 | 6 |
| NaCl | 42 | 7 |
| Cs_3_Sb_2_I_9_ | 42.7 | 8 |
| Rb_3_Sb_2_I_9_ | 50.4 | 8 |
| Cs_2_Ag_0.16_Na_0.84_InCl_6_ | 51.0 | 6 |
| Cs_3_Bi_2_I_9_ | 79.5 | 8 |
| ZnO nanocrystals | 30.8 | This work |


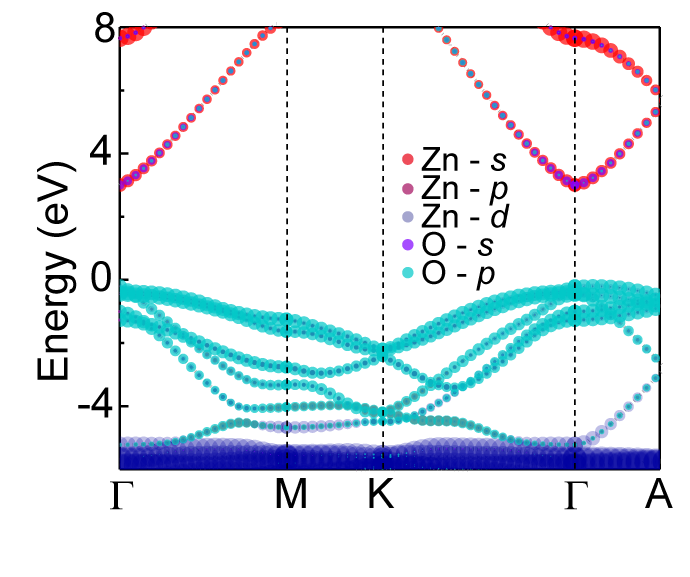


Figure S6**|** Orbit-resolved projected density of states of ZnO with PBE0 functional.


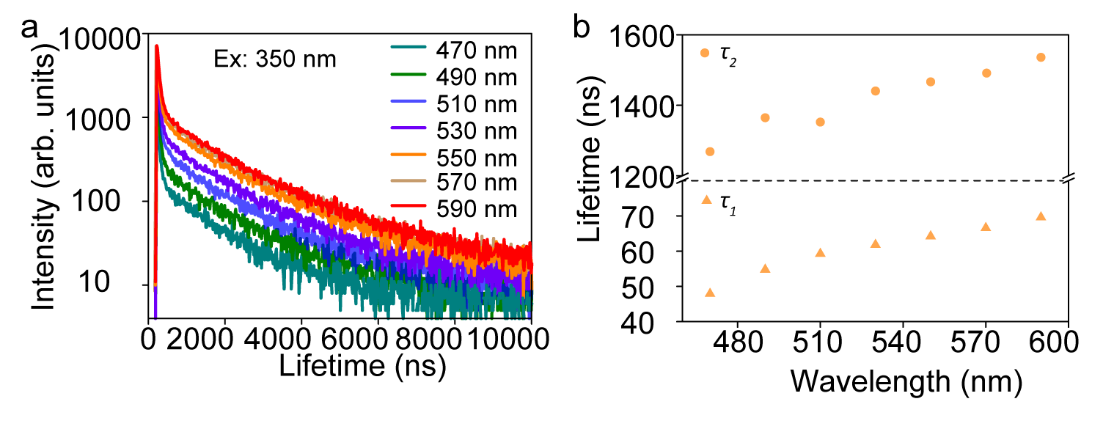


Figure S7**| a**, PL decay curves of ZnO nanocrystals at selected wavelength (470 nm-590 nm) under 350 nm excitation at room temperature. **b**, The PL lifetime changes of ZnO nanocrystals at selected wavelength ((470 nm-590 nm)) under 350 nm excitation.


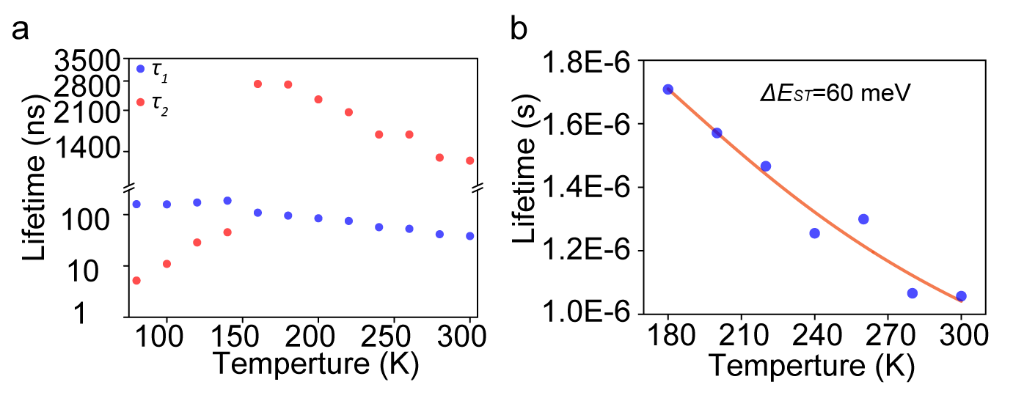


Figure S8**| a**, Temperature-dependent PL lifetime of the ZnO nanocrystals (*τ_1_*: the short lifetime; *τ_2_*: the long lifetime). **b**, The average lifetime of ZnO nanocrystals measured at different temperatures.

Table S3. The angular of ground state and the corresponding configurations.

|  | *S*=0 | Configuration |
| --- | --- | --- |
| *L*=2 | *J*=2 | *^1^D_2_* |
| *L*=1 | *J*=1 | *^1^P_1_* |
| *L*=0 | *J*=0 | *^1^S_0_* |

Table S4**.** The angular of excited state and the corresponding configurations.

|  | *L*=1 | Configuration |
| --- | --- | --- |
| *S*=1 | *J*=2,1,0 | *^3^P_210_* |
| *S*=0 | *J*=1 | *^1^P_1_* |


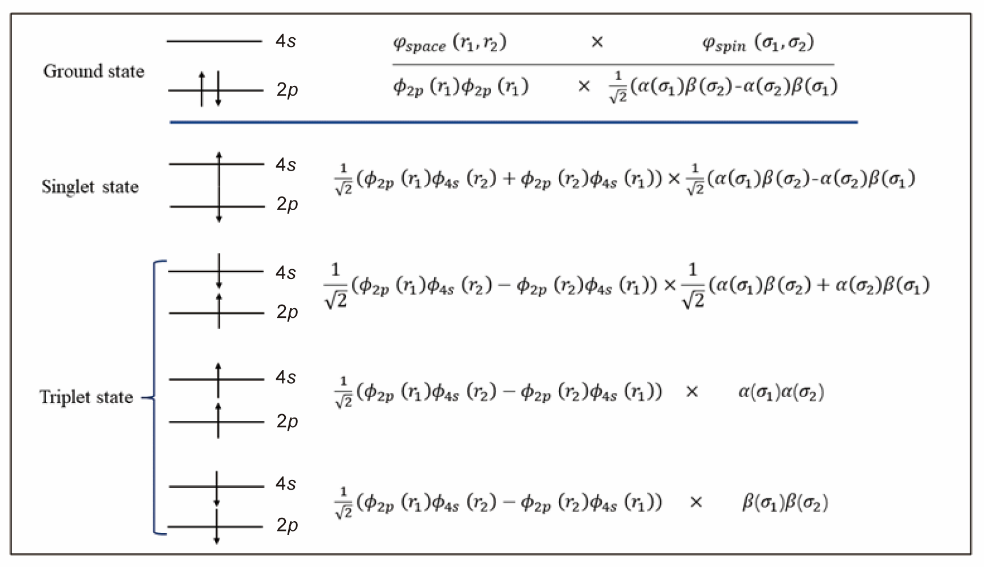


Figure S9**|** The wavefunctions of the excited state. $\phi_{2p}$: Space wavefunction in 2*p* orbit; $\phi_{4s}$: Space wavefunction in 4*s* orbit; $\alpha:$ Spin up wavefunction; $\beta:$ Spin down wavefunction; $\sigma_{1}:$ Particle 1; $\sigma_{2}:$ Particle 2.


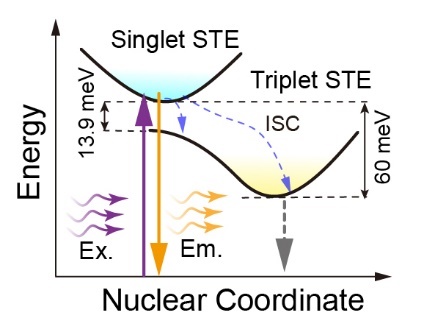


Figure S10**|** The schematic diagram of singlet/triplet mixed STE emission mechanism of the ZnO nanocrystals.


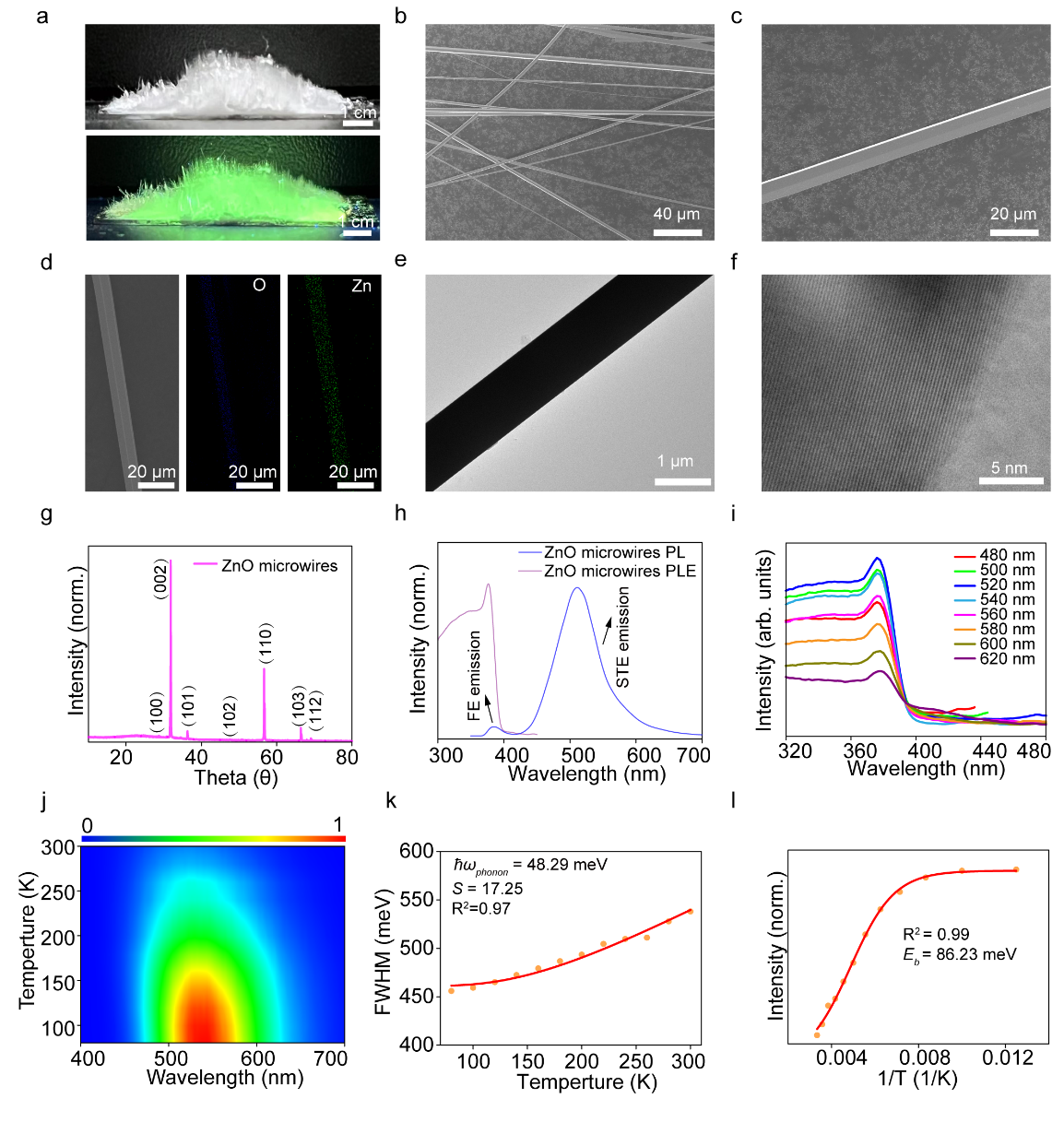


Figure S11**| a**, Digital photos of ZnO microwires under the sunlight and UV light excitation. **b** and **c**, SEM images of the ZnO microwires. **d**, The EDS images of O and Zn element in ZnO microwires. **e**, TEM images of ZnO microwires. **f**, High-resolution TEM images of ZnO microwires. **g**, XRD spectrum of ZnO microwires. **h**, The PLE and PL spectra of ZnO microwires. **i**, The PLE spectra of different emission wavelengths. **j**, Temperature-dependent PL spectra of ZnO microwires (80 K-300 K). **k**, The FWHM of emission spectrum as a function of temperature, and the solid line is the fitting result. **l**, Temperature-dependent integrated STE emission intensity of ZnO microwires.


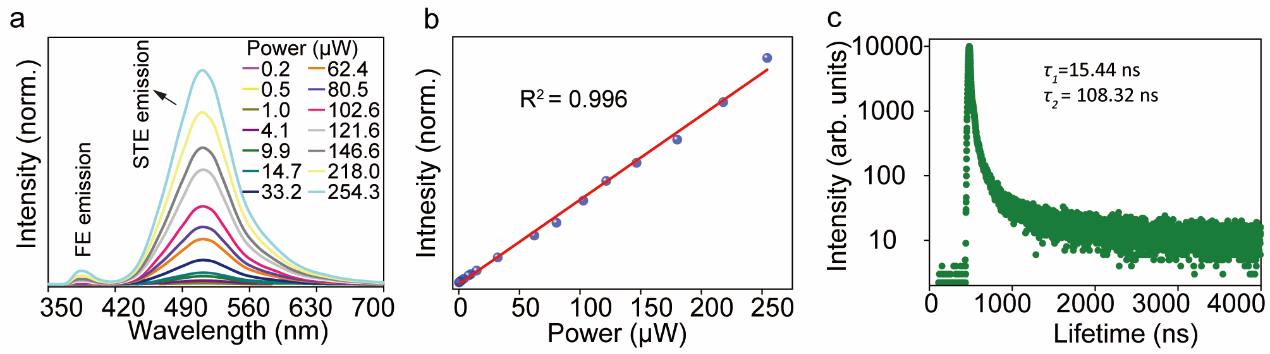


Figure S12**| a**, PL spectra of the ZnO microwires under different power 355nm laser excitation at 300 K (excitation power: 0.2-254.3 μW). **b**, Emission intensity versus excitation power for the ZnO microwires. **c**, The PL decay curve of ZnO microwires.


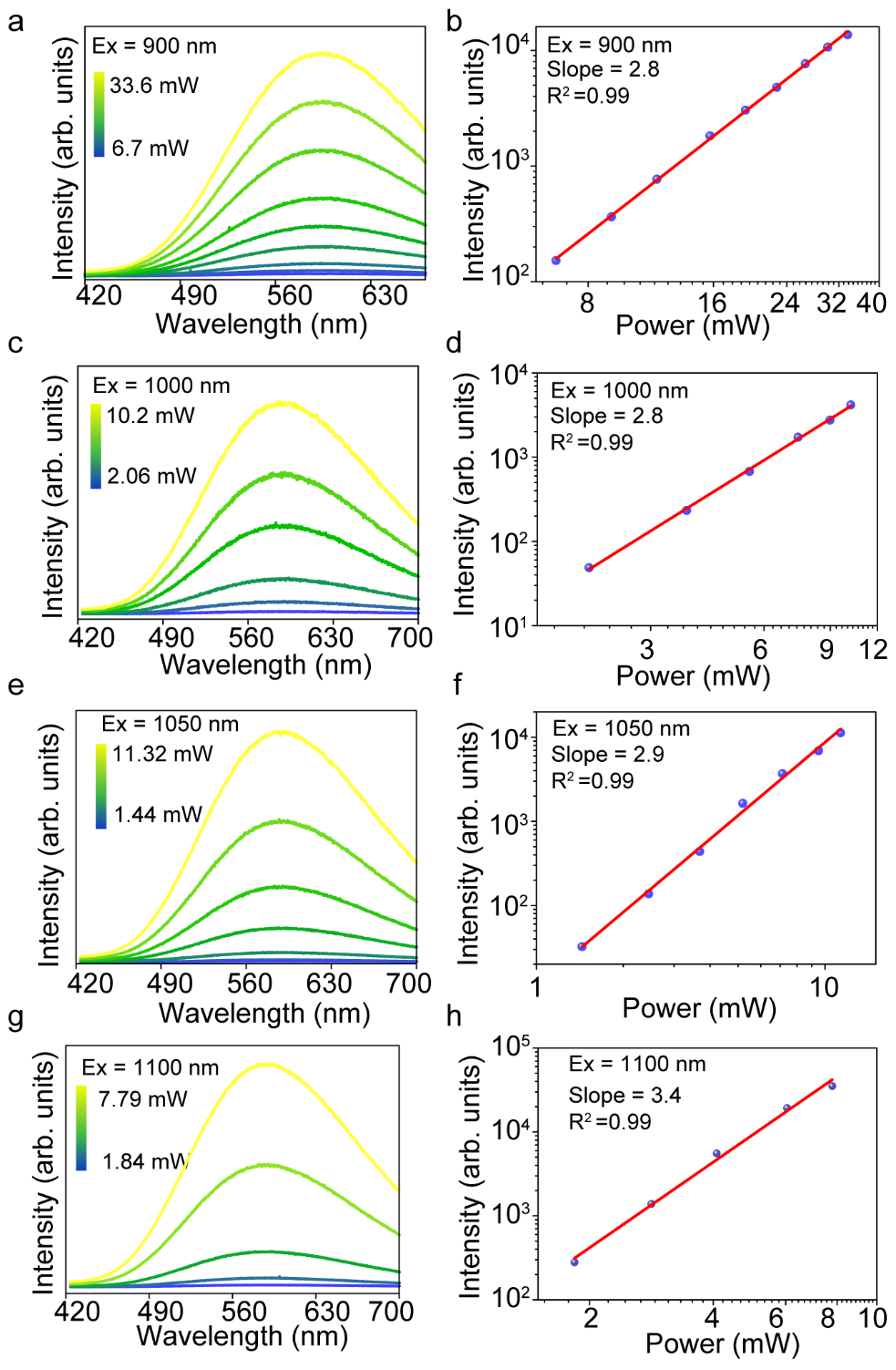


Figure S13**| a**, **c**, **e** and **g**, STE emission spectra of the ZnO nanocrystals under 900 nm, 1000 nm, 1050 nm and 1100 nm fs-laser excitation with different excitation intensities, respectively. **b**, **d**, **f** and **h**, Cubic dependence of the integrated emission intensity in ranges of 450-700 nm on excitation intensity, respectively.


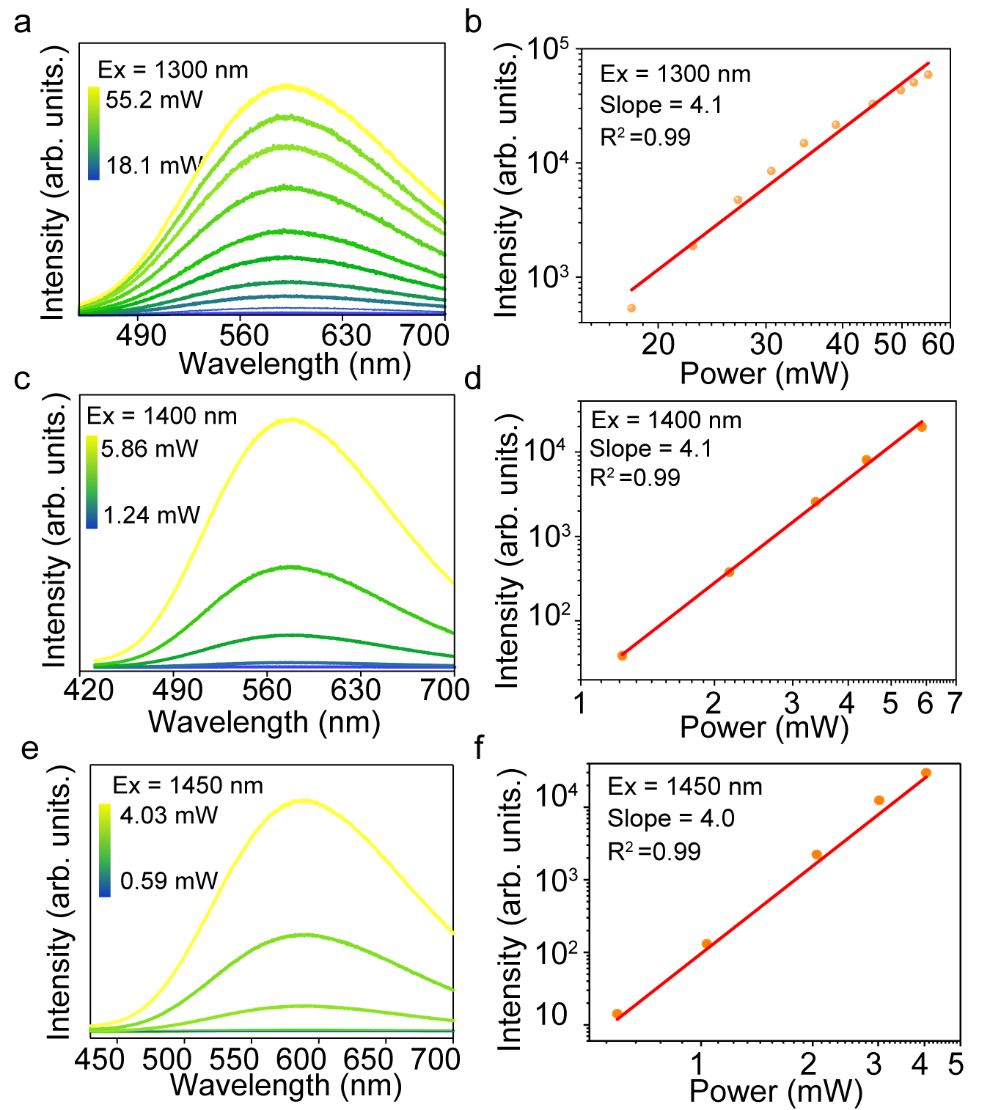


Figure S14**| a**, **c** and **e** and STE emission spectra of the ZnO nanocrystals under 1300 nm, 1400 nm and 1450 nm fs-laser excitation with different excitation intensities, respectively. **b**, **d** and **f**, Quartic dependence of the integrated fluorescence intensity in ranges of 450-700 nm on excitation intensity, respectively.


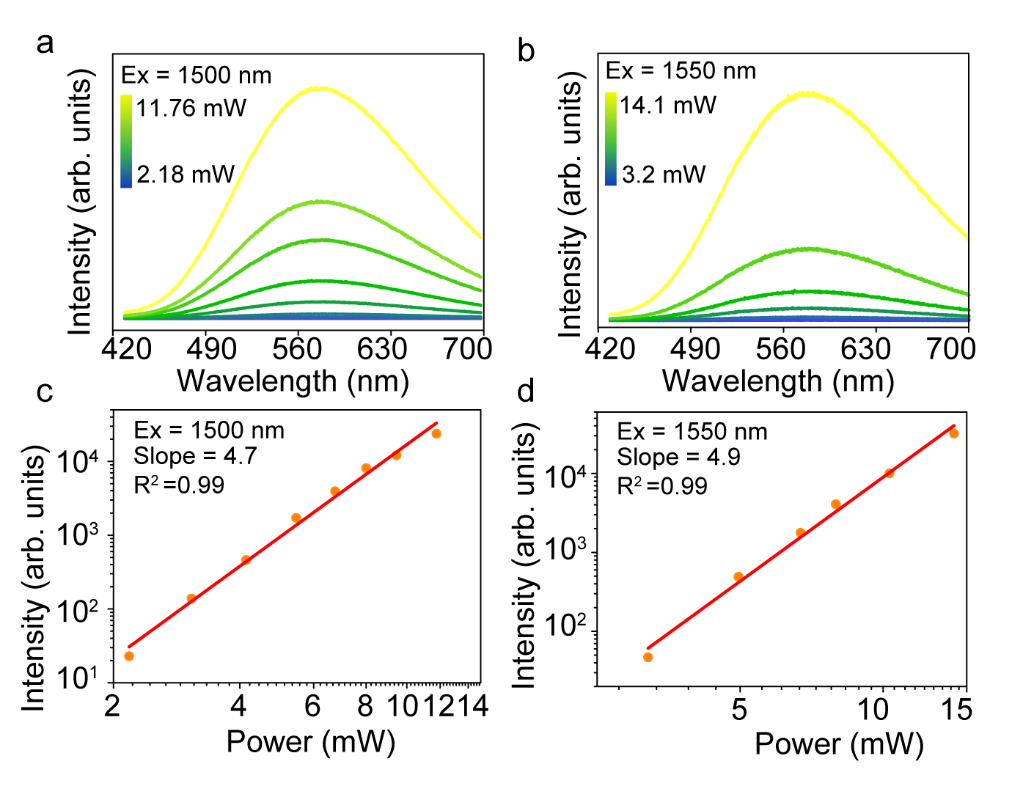


Figure S15**| a** and **b**, STE emission spectra of the ZnO nanocrystals under 1500 nm and 1550 nm fs-laser excitation with different excitation intensities, respectively. **c** and **d**, Quintic dependence of the integrated emission intensity in ranges of 450-700 nm on excitation intensity, respectively.


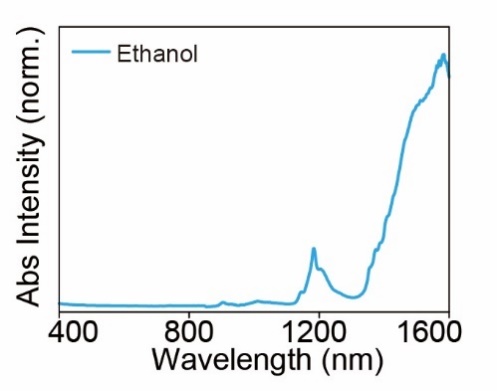


Figure S16**|** Absorption spectrum of ethanol at room temperature.


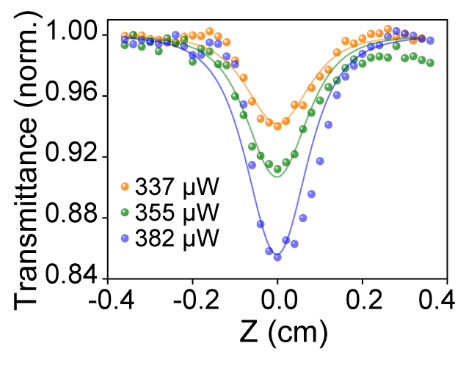


Figure S17**|** Open-aperture Z-Scan experimental curves and theoretically fitted curves of ZnO nanocrystals under 800 nm fs laser excitation.


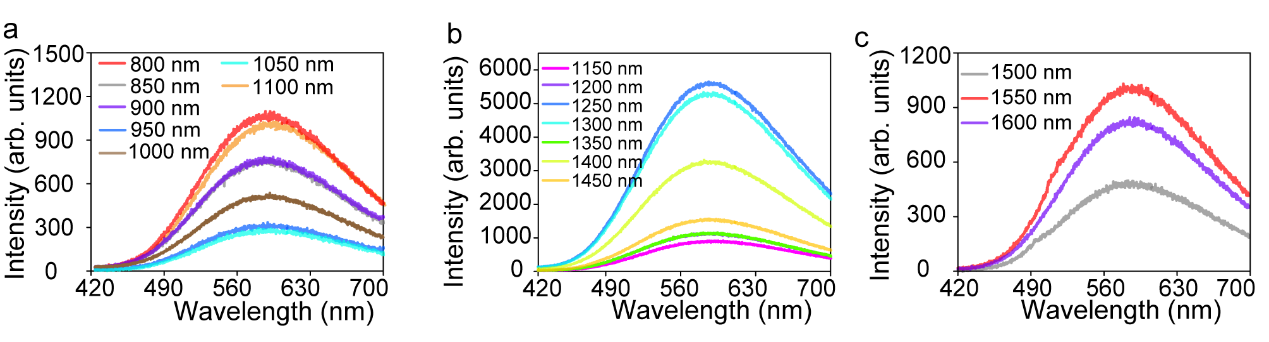


Figure S18**| a**, Three-photon STE emission spectra of ZnO nanocrystals under 800-1100 nm fs-laser excitation with different excitation intensities, respectively. **b**, Four-photon STE emission spectra of ZnO nanocrystals under 1150-1450 nm fs-laser excitation with different excitation intensities, respectively. **c**, Five-photon STE emission spectra of ZnO nanocrystals under 1500-1600 nm fs-laser excitation with different excitation intensities, respectively.


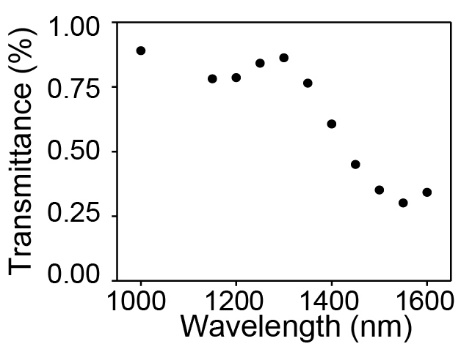


Figure S19**|** The transmittance of the ethanol solvent at different excitation wavelength (1150 nm, 1200 nm, 1250 nm, 1300 nm, 1350 nm, 1400 nm, 1450 nm, 1500 nm, and 1600 nm).

Table S5**.** Comparison of the three-photon absorption cross section of ZnO nanocrystals with the reports values from MAPbBr_3_ nanocrystals, MAPbBr_3_/(OA)_2_PbBr_4_ nanocrystals, CsPbBr_3_ nanocrystals and traditional semiconductor nanocrystals.

| **Materials** | **Excitation laser source (nm)** | **PLQY** | ***σ_3_* (10^-75^ cm^6^ s^2^ photon^-2^)** | **Reference** |
| --- | --- | --- | --- | --- |
| MAPbBr_3_ nanocrystals | 1050-1500 nm, 50 fs, 1 kHz | ~84% | 3.9-33 | 9 |
| MAPbBr_3_/(OA)_2_PbBr_4_ nanocrystals | 1050-1500 nm, 50 fs, 1 kHz | ~92% | 27-240 | 9 |
| CsPbBr_3_ nanocrystals | 1050-1500 nm, 50 fs, 1 kHz | ~55% | 7-140 | 9 |
| ZnSe/ZnS nanocrystals | 1064 nm, 35 ps, 10 Hz | 17% and 22% | 1.4-2.4 | 10 |
| ZnS nanocrystals | 950 nm | 20%-29% | 0.000032-0.16 | 11 |
| ZnO nanocrystals | 800-1100 nm, 50 fs, 1 kHz | 60.5% | 0.00002-0.0007 | This work |

Table S6**.** Comparison of the four-photon absorption cross section of ZnO nanocrystals with the reports values from MAPbBr_3_ nanocrystals, MAPbBr_3_/(OA)_2_PbBr_4_ nanocrystals, CsPbBr_3_ nanocrystals.

| **Materials** | **Excitation laser source (nm)** | **PLQY** | ***σ_4_* (10^-106^ cm^8^ s^3^ photon^-3^)** | **Reference** |
| --- | --- | --- | --- | --- |
| MAPbBr_3_ nanocrystals | 1500-2200 nm, 50 fs, 1 kHz | ~84% | 3.6-300 | 9 |
| MAPbBr_3_/(OA)_2_PbBr_4_ nanocrystals | 1500-2200 nm, 50 fs, 1 kHz | ~92% | 21-2400 | 9 |
| CsPbBr_3_ nanocrystals | 1500-2200 nm, 50 fs, 1 kHz | ~55% | 7.1-690 | 9 |
| ZnO nanocrystals | 1150-1450 nm, 50 fs, 1 kHz | ~60.5% | 0.04-0.48 | This work |

Table S7**.** Comparison of the five-photon absorption cross section of ZnO nanocrystals with the reports values from MAPbBr_3_ nanocrystals, MAPbBr_3_/(OA)_2_PbBr_4_ nanocrystals, CsPbBr_3_ nanocrystals.

| **Materials** | **Excitation laser source (nm)** | **PLQY** | ***σ_5_* (10^-136^ cm^10^ s^4^ photon^-4^)** | **Reference** |
| --- | --- | --- | --- | --- |
| MAPbBr_3_ nanocrystals | 2050-2300 nm, 50 fs, 1 kHz | ~84% | 0.039-2.4 | 9 |
| MAPbBr_3_/(OA)_2_PbBr_4_ nanocrystals | 2050-2300 nm, 50 fs, 1 kHz | ~92% | 0.29-20 | 9 |
| CsPbBr_3_ nanocrystals | 2050-2300 nm, 50 fs, 1 kHz | ~55% | 0.092-6.5 | 9 |
| ZnO nanocrystals | 1500-1600 nm, 50 fs, 1 kHz | ~60.5% | 0.33-6.47 | This work |


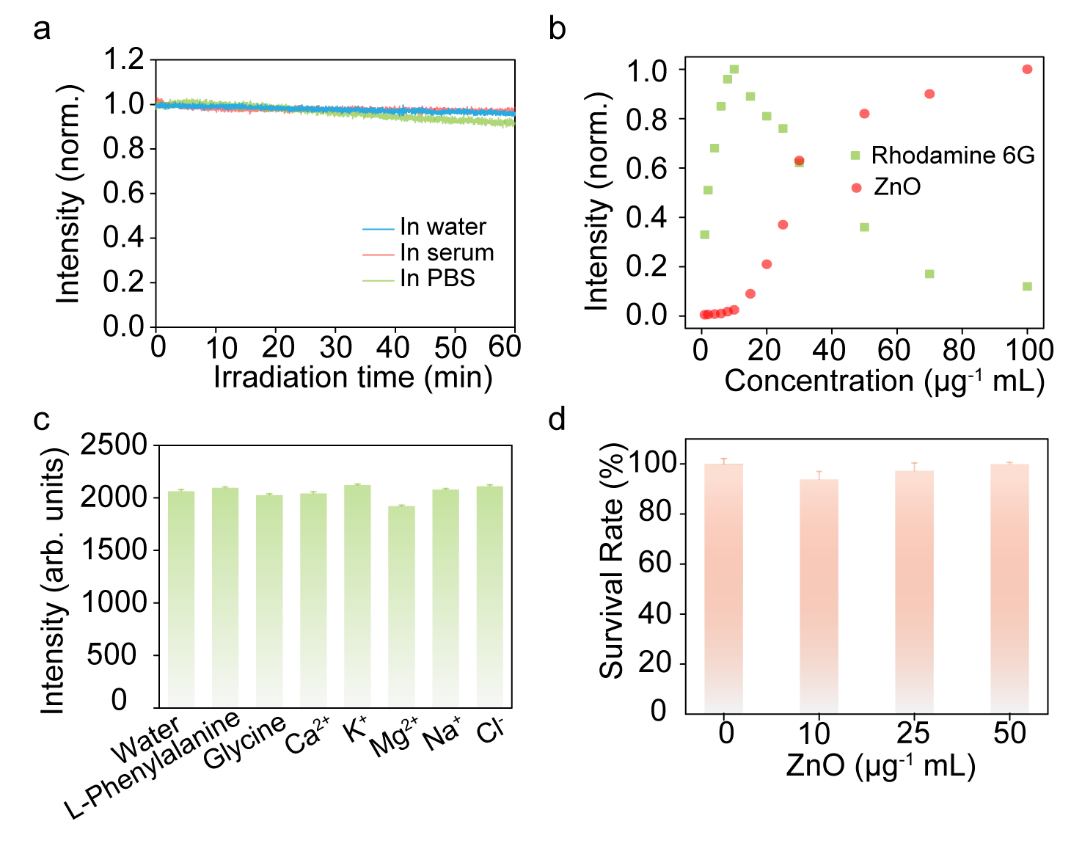


Figure S20**| a**, Photostability of the ZnO nanocrystals in different medias under irradiation of 365 nm for 60 mins. **b**, The emission intensity of ZnO nanocrystals and Rhodamine 6G at different concentrations. **c**, The effect of important amino acids and ions in animals on the emission intensity of the ZnO nanocrystals. Error bars represent ± 1 standard deviation from the mean, m = 3. **d**, Flow cytometry evaluation of the viability of Hella cells exposed to ZnO nanocrystals at various concentrations (0, 10, 25, and 50 μg mL^-1^). Error bars represent ± 1 standard deviation from the mean, m = 3.

High emission efficiency, broad emission spectrum from 400 to 850 nm and capability of being excited by multiphoton, endowing the water-soluble ZnO nanocrystals with great potential for realizing deep-tissue and high-resolution bioimaging. Photostability and biocompatibility as key properties of fluorescent agent have been investigated before exploration of multiphoton bioimaging. The photostability of the ZnO nanocrystals dispersed in water, PBS and serum solution has been evaluated, and the emission intensity decreases slightly (within 5%) after irradiation (365 nm) for 60 min, as shown in Fig. S20a. Important amino acids and ions in vivo do not substantially affect the emission property of the ZnO nanocrystals (Fig. S20b). In addition, the emission intensity of the ZnO nanocrystals proportionally increases with the concentration, indicating potential application for quantitative analysis, as shown in Fig. S20c. Before using ZnO nanocrystals for bioimaging, the nanocrystals influence on Hella cells viability was assessed by CKK-8 methods (Fig. S20d). The cell viability rate reaches 95% cell viability at a nanocrystal concentration of up to 50 μg mL^-1^ during 7 days of culture, which confirms that ZnO has good biocompatibility.


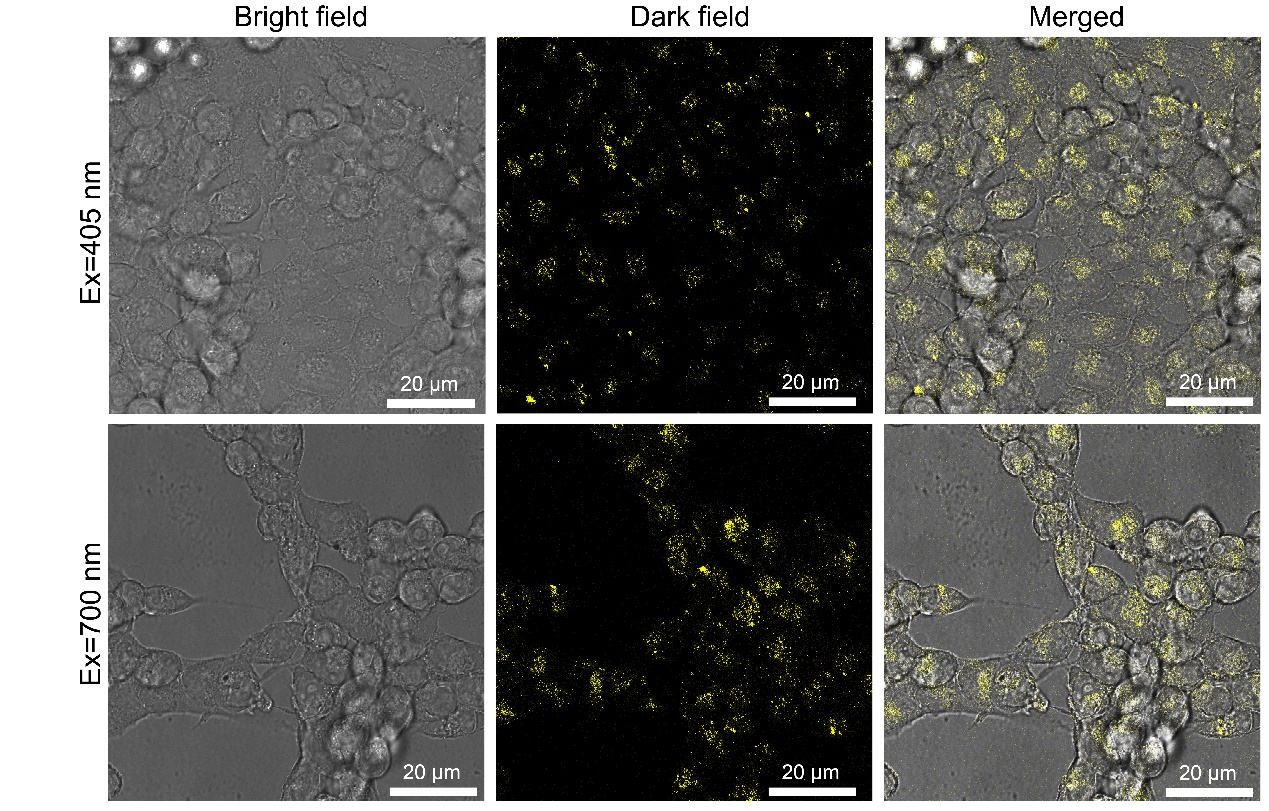


Figure S21**|** One-photon and two-photon emission images of B16-F10 cells treated with 25 μg mL^-1^ ZnO nanoprobe. Different imaging channels are displayed horizontally for each sample (from left to right): bright field, dark field and merged images. The images were collected at 520-590 nm.


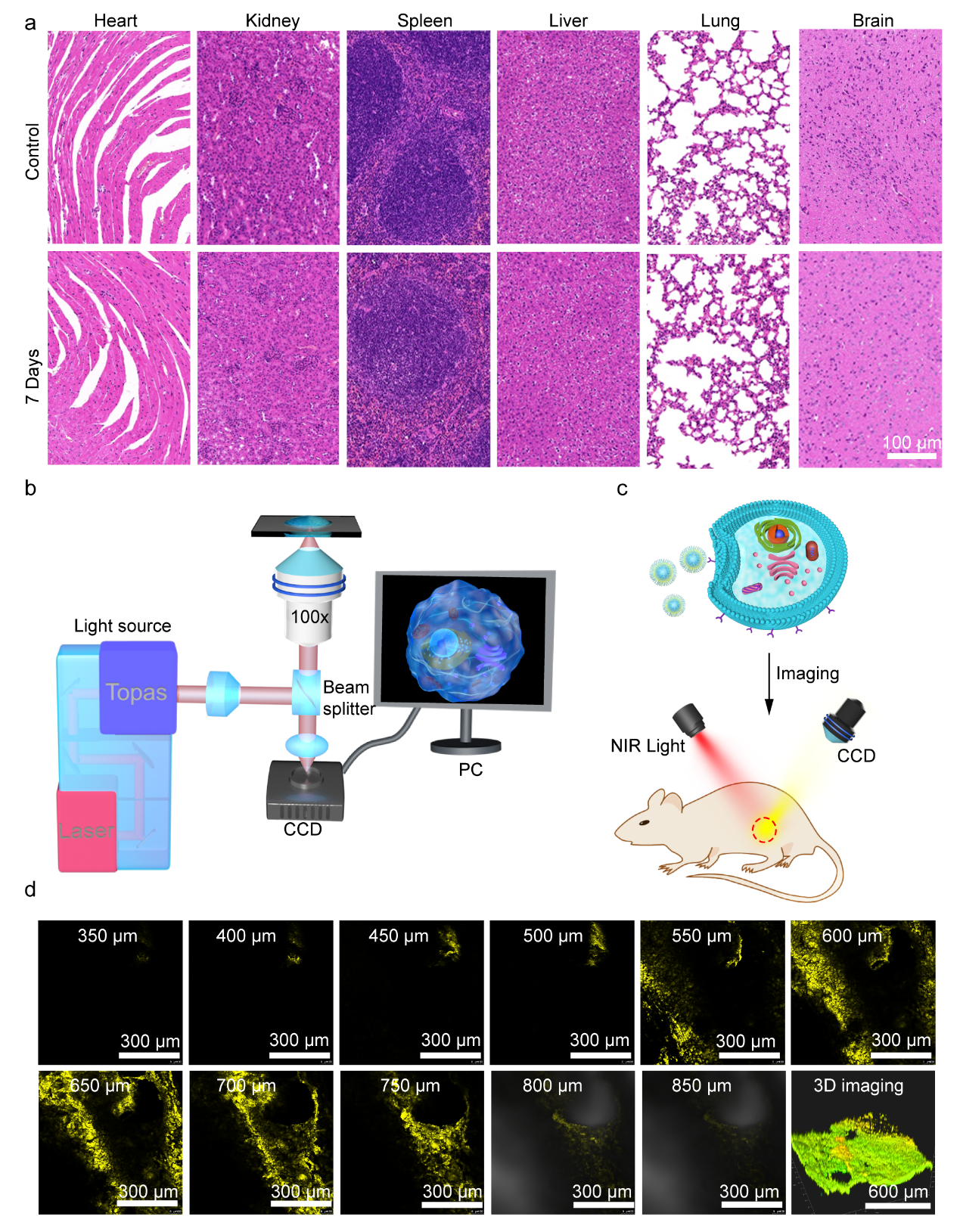


Figure S22**| a**, Histological H&E staining for main organs (lung, liver, brain, spleen, kidneys and heart) of the mice intravenously administrated with PBS and ZnO nanocrystals for 7 days. **b**, Schematic diagram of multiphoton confocal fluorescence imaging system. **c**, Schematic diagram of two-photon fluorescence imaging in vivo. **d**, In vivo, two-photon fluorescence images after the ZnO nanocrystals injection at different focal depths (350 to 850 μm).

The effect on the specific biological tissues has also been evaluated by a histological study. The ZnO nanocrystal aqueous solution were injected into mice as experimental and control groups, and the tissues were sliced and stained by hematoxylin and eosin (H&E) after 7 days, as shown in Fig. S22a. The stained tissues were observed carefully using microscope, and histological analysis reveal that no noticeable damage or inflammatory lesions are observed in major organs (brain, heart, lung, liver, spleen, and kidney) of the mice after treating with ZnO nanocrystals for a long time, suggesting good biocompatibility. The above results confirm that ZnO nanocrystals have low biological toxicity and good photostability, expecting to be used in biological imaging.


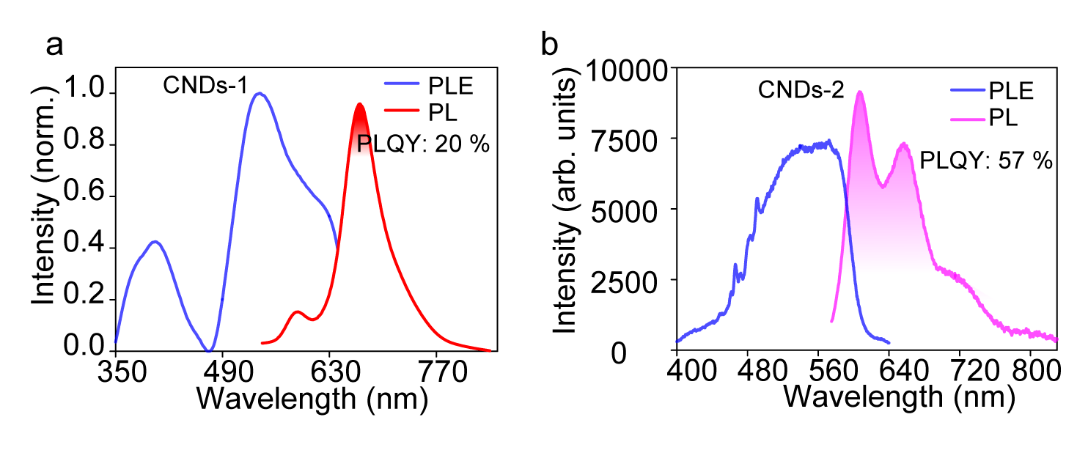


Figure S23**| a,** The PLE and PL spectra of CNDs-1^12^. **b**, The PLE and PL spectra of CNDs-2^13^.


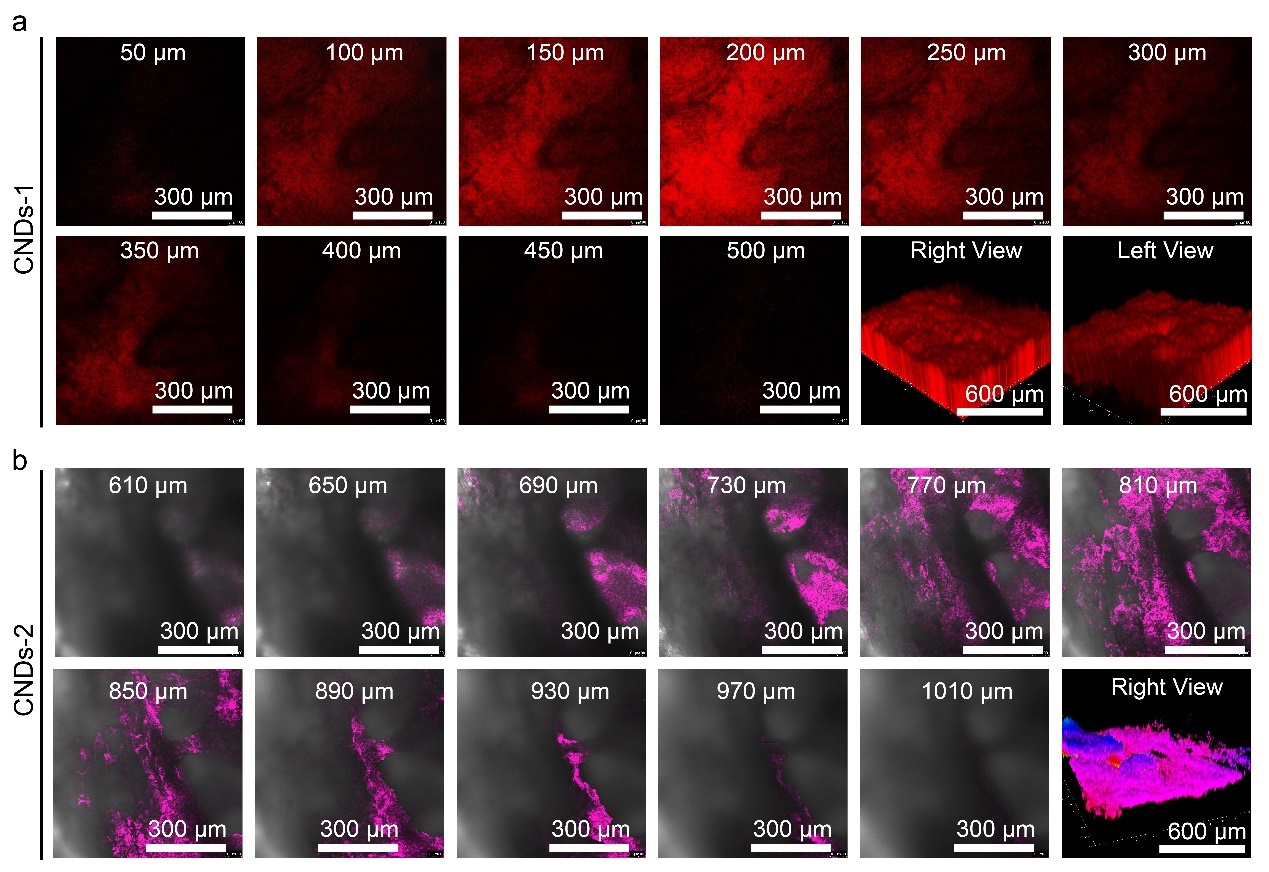


Figure S24**| a**, Two-photon fluorescence images after CNDs-1 injection at different focal depths. **b**, Two-photon fluorescence images after CNDs-2 injection at different focal depths.

Table S8**.** Comparative study of two-photon deep tissue imaging with different materials.

| **Materials** | **Multiphoton imaging** | | **Maximum imaging depth (μm)** | **References** |
| --- | --- | --- | --- | --- |
| Functionalized Acrylonitriles | | Two-photon | 60 | 14 |
| Covalent Organic Framework | | Two-photon | 155 | 15 |
| Small-Molecule Probes | | Two-photon | 170 | 16 |
| Biomimetic Nanoprobes | | Two-photon | 300 | 17 |
| Carbon Nanodots | | Two-photon | 300  440 | 18, 19 |
| Graphene quantum dots | | Two-photon | 320 | 20 |
| Silica Nanoprobe | | Two-photon | 350 | 21 |
| FeSe quantum dots | | Two-photon | 500 |  |
| AIEgens | | Two-photon | 132  424  450  700  800  1065 | 22  23-29 |
| CNDs-1 | | Two-photon | 450 | This work |
| CNDs-2 | | Two-photon | 1010 | This work |
| ZnO nanocrystals | | Two-photon | 850 | This work |

**References**

1. Türck, V. et al. Effect of random field fluctuations on excitonic transitions of individual CdSe quantum dots. *Phys. Rev. B* **61**, 9944-9947 (2000).
2. Zhao, H. & Kalt, H. Energy-dependent Huang-Rhys factor of free excitons. *Phys. Rev. B* **68**, 125309 (2003).
3. Lao, X. Z. et al. Luminescence and thermal behaviors of free and trapped excitons in cesium lead halide perovskite nanosheets. *Nanoscale* **10**, 9949-9956 (2018).
4. Andrews, L. J., Lempicki, A., Mccollum, B. C., Giunta, C. J. & Bartram, R. H. Thermal quenching of chromium photoluminescence in ordered perovskites. I. Temperature dependence of spectra and lifetimes. *Phys. Rev. B* **34**, 2735-2740 (1986).
5. Schulz, M. & Osten, W. V. D. Intensity Dependent Effects in Silver Chloride: Bromine‐Bound Exciton and Biexciton States. *Phys. Status Solidi* **177**, 201-212 (2010).
6. Luo, J. J. et al. Efficient and stable emission of warm-white light from lead-free halide double perovskites. *Nature* **563**, 541-545 (2018).
7. Leung, C. H. & Song, K. S. On the luminescence quenching of F centres in alkali halides. *Solid State Commun.* **33**, 907-910 (1980).
8. Mccal, K. M. et al. Strong Electron-Phonon Coupling and Self-Trapped Excitons in the Defect Halide Perovskites A_3_M_2_I_9_ (A = Cs, Rb; M = Bi, Sb). *Chem. Mater.* **29**, 4129-4145 (2017).
9. Chen, W. Q. et al. Giant five-photon absorption from multidimensional core-shell halide perovskite colloidal nanocrystals. *Nat. Commun.* **8**, 15198 (2017).
10. Lad, A. D., Kiran, P. P., More, D., Kumar, G. R. & Mahamuni, S. Two-photon absorption in ZnSe and ZnSe/ ZnS core/shell quantum structures. *Appl. Phys. Lett.* **92**, 043126 (2008).
11. Yu, J. H. , et al. High-resolution three-photon biomedical imaging using doped ZnS Nanocrystals. *Nature Mater.* **12**, 359-366 (2013).
12. Zhao, W. B. et al, Near-Infrared I/II Emission and Absorption Carbon Dots via Constructing Localized Excited/Charge Transfer State for Multiphoton Imaging and Photothermal Therapy. *Chem. Eng. J.* **452**, 139231 (2023).
13. Liu, K. K. et al. Efficient Red/Near-Infrared-Emissive Carbon Nanodots with Multiphoton Excited Upconversion Fluorescence. *Adv. Sci.* **6**, 1900766 (2019).
14. Niu, G. et al. Functionalized Acrylonitriles with Aggregation-Induced Emission: Structure Tuning by Simple Reaction-Condition Variation, Efficient Red Emission, and Two-Photon Bioimaging. *J. Am. Chem. Soc.* **141**, 15111-15120 (2019).
15. Zeng, J. Y. et al. Covalent Organic Framework for Improving Near-Infrared Light Induced Fluorescence Imaging through Two-Photon Induction. *Angew. Chem. Int. Ed.* **59**, 10087-10094 (2019).
16. Kim, H. M. & Cho, B. R. Small-molecule two-photon probes for bioimaging applications. *Chem. Rev.* **115**, 5014-5055 (2015).
17. Lv, Y. et al. Cancer Cell Membrane-Biomimetic Nanoprobes with Two-Photon Excitation and Near-Infrared Emission for Intravital Tumor Fluorescence Imaging. *ACS Nano* **12**, 1350-1358 (2018).
18. Lesani, P. et al. Two-Photon Dual-Emissive Carbon Dot-Based Probe: Deep-Tissue Imaging and Ultrasensitive Sensing of Intracellular Ferric Ions. *ACS Appl. Mater. Inter.* **12**, 18395-18406 (2020).
19. Yuan, F. L. et al. Nitrogen-Rich D-π-A Structural Carbon Quantum Dots with a Bright Two-Photon Fluorescence for Deep-Tissue Imaging. *ACS Appl. Bio Mater.* **1**, 853-858 (2018).
20. Meng, H. M., Zhao, D., Li, N. & Chang, J. A graphene quantum dot-based multifunctional two-photon nanoprobe for the detection and imaging of intracellular glutathione and enhanced photodynamic therapy. *Analyst* **143**, 4967-4973 (2018).
21. Wang, X. H. et al. Folate receptor-targeted aggregation-enhanced near-IR emitting silica nanoprobe for one-photon in vivo and two-photon ex vivo fluorescence bioimaging. *Bioconjugate Chem.* **22**, 1438-1450 (2011).
22. Kwon, J. et al. FeSe quantum dots for in vivo multiphoton biomedical imaging. *Sci. Adv.* **5**, eaay0044 (2019).
23. Ding, D. et al. Ultrabright organic dots with aggregation-induced emission characteristics for real-time two-photon intravital vasculature imaging. *Adv. Mater.* **25**, 6083-6088 (2013).
24. Li, Y. Y. et al. ACQ-to-AIE Transformation: Tuning Molecular Packing by Regioisomerization for Two-photon NIR Bioimaging. *Angew. Chem.* **132**, 12922-12926 (2020).
25. Qi, J. et al. Aggregation-Induced Emission Luminogen with Near-Infrared-II Excitation and Near-Infrared-I Emission for Ultradeep Intravital Two-Photon Microscopy. *ACS Nano* **12**, 7936-7945 (2018).
26. Zheng, Z. et al. Aggregation-Induced Nonlinear Optical Effects of AIEgen Nanocrystals for Ultradeep In Vivo Bioimaging. *Adv. Mater.* **31**, e1904799 (2019).
27. Qi, J. et al. Real-Time and High-Resolution Bioimaging with Bright Aggregation-Induced Emission Dots in Short-Wave Infrared Region. *Adv. Mater.* **30**, e1706856 (2018).
28. Zong, L. Y. et al. Tunable Aggregation-Induced Emission Nanoparticles by Varying Isolation Groups in Perylene Diimide Derivatives and Application in Three-Photon Fluorescence Bioimaging. *ACS Nano* **12**, 9532-9540 (2018).
29. Lou, X., Zhao, Z. & Tang, B. Z. Organic Dots Based on AIEgens for Two-Photon Fluorescence Bioimaging. *Small* **12**, 6430-6450 (2016).
